# Moderation and Mediation Analysis of the Relationship between Total Protein Concentration and the Risk of Depressive Disorders in Older Adults with Function Dependence in Home Care

**DOI:** 10.3390/nu10101374

**Published:** 2018-09-26

**Authors:** Grzegorz Józef Nowicki, Barbara Ślusarska, Agnieszka Bartoszek, Katarzyna Kocka, Alina Deluga, Hanna Kachaniuk, Marta Łuczyk

**Affiliations:** 1 Department of Family Medicine and Community Nursing, Medical University of Lublin, Staszica 6, 20-081 Lublin, Poland; basiaslusarska@gmail.com (B.Ś.); agabartoszek@wp.pl (A.B.); katarzyna48@op.pl (K.K.); alina_deluga@poczta.onet.pl (A.D.); hakach@op.pl (H.K.); 2 Department of Oncology, Medical University of Lublin, Staszica 6, 20-081 Lublin, Poland; luczykmarta@op.pl

**Keywords:** depression, total protein, elderly people, physical function, long-term care

## Abstract

Due to its devastating consequences, late-life depression is an important public health problem. The aim of the study was an analysis of variables which may potentially influence the risk of depression (GDS-SF). Furthermore, the aim was to study possible mediating effects of given variables on the relationship between the total protein concentration and the risk of depression in older adults with chronic diseases, and physical function impairment. The research sample included 132 older adults with chronic conditions and physical function impairments, residing in a long-term care in residential environment. In the studied group of sensory organs, diseases proved to be a significant moderator of the relationship between GDS-SF and total serum protein concentration. A stronger relationship was observed in subjects suffering from diseases of sensory organs (b = −6.42, 95% CI= −11.27; −1.58). The Barthel index and 25(OH)D vitamin were the most significant mediators of the examined relationship. Cohort research is suggested to confirm the hypothesis.

## 1. Introduction

Functional status, understood as the ability to perform the basic and instrumental activities of daily living (ADL), is of key importance for assessing quality of life (QOF) in older adults, as well as in younger people, in the case of health deficits that lead to a deterioration in functionality. Research into mortality predictors demonstrates that the functional status of elderly people is an independent predictive factor for mortality [1,2,3].

Physical function impairment in patients is a particularly problematic issue in long-term domiciliary care, and may lead to numerous health problems and result in difficulties when providing homecare. Nursing homecare is becoming a significant sector in the healthcare system for older adults. Long-term nursing care is of great importance for this healthcare system. A systematic review of long-term care in Europe, including data from 18 countries, indicates a wide variety of long-term care systems in individual countries, while also painting a picture of limited access to this form of care for existing needs [4]. In Poland, long-term nursing care is a relatively new and developing discipline. In 1999, residential long-term care facilities, “i.e., nursing homes and care homes for the chronically ill, and incapacitated”, were established. In 2004, medical services provided at homes, under the name of long-term nursing care [5], began to be contracted. Long-term nursing care can be provided only to patients scoring 0–40 points on the Barthel index, along with a referral from a doctor working within a health insurance scheme [6]. Other than the above, the elderly with physical function impairments, who reside in a family environment, are under the care of informal carers (usually family members), who support patients with slight or moderate dependence on the Barthel index.

The results of the SHARE research project conducted during 2010–2011 show that the proportion of older adults with functional limitations, which is a basic element determining the demand for certain forms of assistance in daily life, is high in Poland. Nearly 20% of people aged 65–79 years, and over 40% of individuals aged over 80 years, declare problems with performing daily living activities (ADL). High percentages of the elderly (23% of individuals aged 65–79 years, and 54% of individuals over 80 years old) declare problems with performing instrumental daily living activities (IADL) [7].

Due to the significant role of long-term care, the demand for such care is high. However, the supply of long-term care in Poland is mainly informal, and is organized by the family—the care is provided primarily by the closest family members in the patient’s home. Several independent estimations consistently indicate that most elderly people (between 80% [8] and 93% [9]) are under informal care. This high supply of informal care results from traditional family relationships. Frequently, older adults reside with their children (high cohabitation index).

In a Polish literature review, there are few estimations of the quantity and functional status of individuals. When patients with physical functional impairment in long-term care were examined, using the Barthel index, the group (*n* = 296) included 21.3% of the examined scoring 0 points, and 78.7% of the examined scoring 5–40 [5,10]. In another study concerning the assessment of the functional status of patients with long-term domiciliary care, using the Barthel index (*n* = 190), almost two-thirds of the examined (64.21%) were patients with total dependence (Barthel index 0–20), who required considerable help in performing daily life activities or were unable to provide self-care. The remaining patients (35.79%) were characterized by moderate dependence, scoring 21–40 on the Barthel index [11]. In a cumulative assessment of patients receiving long-term nursing care (*n* = 575) during 2004–2008 in northeastern Poland, 45% (*n* = 259) of people scored 0 points on the Barthel index. The highest value of points qualifying for long-term nursing care on the Barthel scale (40 points) was found in 4.9% (*n* = 28) of the examined individuals. The remainder of the individuals, 50.1% (*n* = 288), scored 5–35 points, and 1–5 on the Barthel index, in the assessment of patients’ self-care [12].

Physical function impairment and resulting mobility limitations are the reasons for impairments to the physiological and biochemical reactions of human organism. The functions of cardiovascular, respiratory, nervous, and muscular-bone systems, along with water–electrolyte balance, mainly calcium metabolism, are distorted, affecting blood clotting. The tempo of energy conversion is also changed, and organism homeostasis is distorted [13]. Hypokinesis changes body composition, protein metabolism, hormonal profile and muscle function [14,15,16]. Additionally, hypokinesis results in a decrease in the production of certain neurotransmitters and neuromodulators in the brain, e.g., noradrenaline and endorphins. Despondence might occur as well as lower adaptation capacities of the central nervous system (concentration difficulties, sleep disorders, anxiety, aggression, as well as depression) [17].

Malnutrition constitutes an important and increasingly frequently diagnosed problem in patients over the age of 65. Age-related involutional senile changes, coexisting polypathology and polypragmasia, poor general and eating habits, and unfavorable social and economic conditions, as well as cultural conditions all contribute to significant dietary abnormalities [18]. Epidemiological data on malnutrition and nutrient content in the diet (including the calorific-protein composition of food intake) among elderly people in Poland living in long-term domiciliary care are insufficient. This is, among other reasons, due to the lack of a gold standard for assessing nutritional status [19,20], as well as their time-consuming implementation. Polish epidemiological studies assessing malnutrition in people over 65 years of age cover the entire group of such respondents, without a focus on the division of patients in long-term domiciliary care. Multi-center research conducted by WOBASZ Senior among a representative group of 1013 elderly using a MNA (Mini Nutritional Assessment) test demonstrated that 13% of seniors were undernourished and 57% were at risk of malnutrition [21]. Studies conducted, collected and averaged in different countries up to 2006 have proved that the malnutrition assessed with the MNA scale was most commonly found in hospitalized patients (23%), compared to residents of nursing homes (21%). However, the risk of malnutrition was higher in patients of social care homes (51%) than in those in hospital patients (46%). Older people living in their own homes were characterized as having the lowest severity of undernutrition (2%) and the lowest risk of malnutrition (24%) [22]. Following malnutrition, there is a series of adverse changes in all organs and systems of the elderly human body. Weight loss, muscle weakness and a decrease in psychomotor performance have all been observed [23]. In the gastrointestinal tract, there are functional disorders such as weakness of intestinal motility, digestive disorders and absorption, colonization of the small intestine with bacteria, fatty liver and reduced protein synthesis, reduction of pancreatic mass and secretion of digestive enzymes. Adverse consequences also apply to the respiratory system (atrophy of the respiratory muscles with subsequent deterioration of ventilation efficiency and greater predisposition to pneumonia), cardiovascular system (impaired systolic function of the myocardium) or the skeletal system (increased risk of osteoporosis). Nutritional deficiencies cause the appearance of nutritional deficiency anemia and disorders in fluid and electrolyte balance. There is an impairment of cellular and humoral immunity (resulting in increased frequency of infections), and a decrease in serum protein concentration (resulting in impaired wound healing and an increased risk of pressure ulcers in bed-ridden patients). Patients are less responsive to treatment and more often impacted by perioperative complications. The time of their treatment/recovery is longer, which inherently increases the cost of therapy [24]. The risk of disease and death increases, as well as the risk of institutionalization, resulting in the quality of life of older patients worsening [25,26,27,28]. The research concerning factors affecting recovery or factors maintaining the condition of patients with chronic diseases, and physical functional impairment, for individuals who are under the care of an informal carer and long-term care, should be a priority for researchers interested in long-term domiciliary care.

The aim of the study was an analysis of variables (medical, biochemical, and anthropometric) which may potentially influence the risk of depression. Furthermore, the aim was to study possible mediating effects of given variables on the relationship between the total protein concentration and the risk of depression (GDS-SF) in older adults with chronic diseases, and physical function impairment.

## 2. Materials and Methods

### 2.1. Study Design

The study included 149 patients receiving long-term domiciliary care in 5 facilities (4 in urban areas, and 1 in a rural area) and it was conducted between September 2016 and February 2017. Information was collected through a direct interview, by nurses working in long-term domiciliary care, who were professionally assisting the families of the patients during the domiciliary visits. The nurses (*n* = 18) providing the long-term domiciliary care were acquainted with the study project materials for the laboratory tests and they were prepared for data collection. Data were collected in accordance with the established procedure in the study. On the first day, the nurse filled in the questionnaire with the patient, collected measurements, and informed the patient about the preparation for the laboratory tests to be taken on the following day (blood collection in the morning, in fasted state, after no food intake for 8–12 h). On the second day, the patient’s venous blood was collected to mark biochemical parameters. Blood was transported in special containers to the laboratory within 30–60 min of the exact collection time.

Inclusion criteria were as follows: low risk associated with the state of nutrition NRS-2002 < 3 (the Nutrition Risk Screening 2002), physical function impairment on the Barthel index of 0–85 points, medical diagnosis of systemic atherosclerosis (ICD10-I70), lack of coexisting carcinoma or renal insufficiency, and lack of cognitive function impairments measured by MMSE ≥ 7 (the Mini-Mental State Examination), as well as the patient being cared for in domiciliary conditions. Consequently, 149 people qualified for the study. However, after conducting a detailed analysis of the collected materials in the questionnaire (due to its completeness), the sample for analysis consisted of 132 people who provided complete information.

Written consent was collected from the managers and directors of the health facilities where the nurses performing research were employed. Each patient was verbally informed by the nurses regarding the objective of the study, and the scope of the questionnaires, measurements, and then written consent was received from the patients. The study was voluntary and anonymous. Nurses completed the questionnaires during a patient interview, and provided the patient with necessary information and explanations. During each stage of the data collection, patients could resign or refuse further participation in the study.

The implemented study procedure was approved by the Bioethics Committee of the Medical University in Lublin (No. KE-0254/13./2016), and was consistent with the Declaration of Helsinki. The study was financed by the Medical University of Lublin.

### 2.2. Measurements

Research tools consisted of two questionnaires: Geriatric Depression Scale-Short Form (GDS-SF) and the Barthel index. Questionnaires were supplemented by a sheet collecting sociodemographic information, and medical interview data. Anthropometric measures were also taken, along with venous blood for laboratory tests.

**Geriatric Depression Scale Short Form (GDS-SF).** The Polish version of the GDS-SF scale [29] was used to measure the degree of risk of incidence of depression symptoms. The GDS-SF scale was created as a screening tool to diagnose the intensity of depression symptoms. The shortened version of the scale consists of 15 statements describing depression symptoms, the examined person confirms or denies their presence (yes/no) during the past two weeks. Interpretation of the shortened version is based on the number of points scored: 0–5. no risk of depression; and 6–15, there exists a risk of depression symptoms. Cronbach’s alpha demonstrated a reliability of 0.86.

**The Barthel index.** The assessment concerning ADL was conducted using the Barthel index. The index takes the following into consideration: meal consumption, mobility, maintenance of personal hygiene, using the lavatory, taking full body baths, mobility on flat surfaces, walking up/down stairs, dressing, and bladder and bowel control. Depending on the range of the patient’s self-care, the patient may score between 0 and 100 points [30]. The amount of scored points shows the degree of resilience to disability and care [31]. The Polish version of the Barthel index showed solid reliability (Cronbach’s alpha ranged from 0.78 to 0.89 with a test-retest *r* ranging from 0.93 to 0.95) [32].

**Demographic and clinical variables**. Collected data describing the sociodemographic aspects of the group included gender, age, marital status (“single” or “in a relationship”), and living arrangements (“alone” or “with family”). Variables taken into consideration when assessing comorbidities, such as systemic atherosclerosis and diseases (or lack thereof) included cardiovascular, respiratory, endocrine, neurological, sensory, mental, rheumatic, and other systems, as well as medicinal substance intakes and their amounts. At the end of this part of the questionnaire, the interviewer calculated the number of years the patient had been under long-term care.

### 2.3. Anthropometric Measurements

As it was not possible to measure patients’ height and weight in domiciliary conditions (bed-ridden patients) or to perform anthropometric assessments, the following criteria were noted: calf, arm, foot–knee length, as well as skinfold under the shoulder and the skinfold of arm triceps muscle.

*Circumference of calf* was measured with an accuracy of up to 0.1 cm in the supine position using an inelastic measuring tape. The tape was placed around a calf without compressing the subcutaneous tissue and was moved along the calf length to get the maximal circumference. The measures of both calves were registered as an average, resulting from the measurements of two trials for each limb, which were combined to establish an average for both legs.

*Mid-upper arm circumference* (MUAC) was measured at the midway point between the olecranon process of the ulna and the acromion process of the scapula [33].

*The length of shin bones* was measured between the distal end of the medial condyle of the tibia, and the apex of the medial malleolus. The location of the anthropometer was parallel to the long axis of the tibia [34].

*Skinfold thickness* (SFT) under the shoulder was measured using a Harpenden Caliperat precisely chosen points. Prior to use, the caliper was thoroughly calibrated to check for strains using an object with known dimensions.

*Triceps skinfold* (TSF) was measured atthe middle point of the non-dominant arm, with the upper limb freely stretched along the body. Next, the skinfold was pinched between the fingers, and the caliper was used. The measurement was performed twice, and the average was used for further analysis.

### 2.4. Blood Collection and Biochemical Analysis

Blood samples were collected in fasting state, after all night rest and a moderately-sized supper the previous day. Blood was collected to a tube containing a clotting activator and a separation substance (granulate). Plasma was separated using centrifugation of 3000 spins per minute, for 10 min. Centrifuged serum was used to mark the levels of biochemical parameters. Total protein concentration, total cholesterol, triglyceride, and high-density lipoprotein cholesterol (HDL) cholesterol were marked using an Advia1800 Siemens apparatus (Siemens Healthcare, Poland, Warsaw), with original reagents. low-density lipoprotein cholesterol (LDL) cholesterol was measured with Friedewald formula (Siemens Healthcare, Poland, Warsaw). We marked the D3 25(OH)D vitamin on a Cobas E411 Roche apparatus (Roche Diagnostics Poland, Warsaw), using an original set of reagents and the electrochemiluminescence method. Both B12 vitamin and folic acid levels were marked using an Advia Centaur Siemens apparatus (Siemens Healthcare, Poland, Warsaw), and the chemiluminescence method.

### 2.5. Statistical Analysis

Categorical variables were reported as numbers and percentages. The distributions of quantitative variables were described by the mean value (M) and the standard deviation (SD) or median (Me), and lower (Q1) and upper quartiles (Q3). The Shapiro–Wilko test was used to assess conformity with a normal distribution.

Linear regression was used to assess the relationship between the values on the scale of depression risk (GDS-SF) and considered variables (age, gender, results of anthropometric measurements, laboratory tests and comorbidities). To find out whether age, gender, or examined comorbidities change the direction or the magnitude of the relationship between total protein concentration and the risk of depression, models with the interaction term between the total protein concentration and the considered variable (moderator) were performed. The significance of the given moderator was assessed according to the change of proportion concerning the explained variance (ΔR^2^).

**Figure 1:**
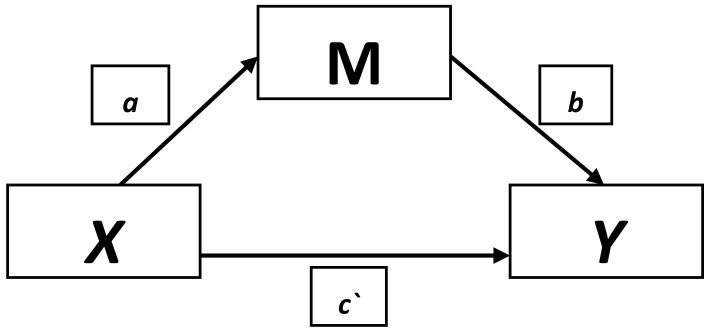
Model presenting the examined mediation effect. X, independent variable (protein concentration); M, mediator; Y, dependent variable (GDS-SF); a, association between X and M; b, association between M and Y after controlling for X; c`, direct effects.

To investigate the mechanism of the relationship between total protein concentration and the risk of depression, mediation analysis was used, taking into consideration the third variable (i.e., mediator). The scheme of the applied mediation analysis is shown in Figure 1. The process macro procedure was used [35]. Several models (steps) of linear regression were performed. The results of the mediation analysis were presented by giving three factors (effects) from models:

1. Providing the coefficient for a given mediator (after controlling for total protein concentration) with 95% confidence interval (95% CI), path b.
2. Direct effect (c`), coefficient of regression model for the total protein concentration after controlling for mediating variable (mediator) with 95% CI.
3. Indirect effect with 95% CI, calculated taking into consideration “bias-corrected” and “accelerated” corrections. The effect is the product (a*b, on the attached scheme) of the coefficients (in regression model) between the total protein concentration and the studied mediating variable which examines the relationship between the coefficient (in the regression model) between the mediating variable and depression.

To establish the significance of the mediation effect, bootstrapping method was used (made for 5000 drawings). This procedure estimates the indirect effect using the bootstrapping technique (generating empirical representation of the sample distribution, treated as a population representation). All analyses were made with IBM SPPS v.22 software (Armonk, NY, USA); as a statistical significance for bilateral tests α = 0.05 was applied.

## 3. Results

### 3.1. Characteristics of the Studied Groups According to Sociodemographic and Medical Data

Table 1 presents characteristics of the studied group. The analysis included only 132 individuals receiving long-term domiciliary care. The average age in the studied group was 78.75 years (SD = 8.75). The majority of the group were women (*n* = 103, 78%) and unmarried (*n* = 83, 62.9%). As many as 118 people (79.2%) lived with their families. The average assessment of physical function (Barthel index) was 43.20 (SD = 27.06). The largest group of people (*n* = 60, 45.5%) scored between 21 and 40 points on the Barthel index, and the second-largest group of people (*n* = 44, 33.3%) scored between 0 and 20 points on the scale. The third-largest group of people (*n* = 28, 21.2%) scored between 41 and 85 points on the Barthel index. All patients were in long-term homecare, which they had used on average for 3.59 years (SD = 2.68). The average amount of prescription medications taken by the patients was 7.9 (SD = 2.8). Patients were characterized by comorbidities, with the most common conditions being rheumatic (77.9%), sensory (65.1%), psychiatric (51.7%), and endocrine (45%) diseases, as well as conditions of the nervous system (26.5%).

In the assessment concerning the risk of the occurrence of depression symptoms, according to GDS-SF, the average was 7.34 points (SD = 3.10). Lack of depression risk (0–5 points) occurred in 25% (*n* = 33) of the sample, and there was a risk of depressive disorders in the other 75% (*n* = 99) of the sample.

Among the respondents, 38 (28.8%) had a total protein concentration within 5.56–6.61 mg/dL; 66 (50%) within 6.62–7.37 mg/dL; and 28% within 7.38–8.14 mg/dL.

### 3.2. Relationship between GDS-SF and Studied Criteria

Table 2 shows results representing a simple linear regression. Negative linear associations were observed between the values on the GDS-SF scale, measuring depression, and the following variables: the results on the modified Barthel index, total protein concentration, concentration of HDL cholesterol, and MUAC. The values on the Barthel index increased as values for the other variables increased. When the Barthel index, total protein, HDL cholesterol and MUAC increase, the values on the GDS-SF decrease.

### 3.3. Moderation Model

Table 3 presents the results concerning testing moderators of relationships between total protein concentration and the value of the GDS-SF scale, which is equal to the occurrence of interactions between total protein and the examined moderators in predicting depression. The estimates of the linear regression models are presented (separately for each moderator): Model 1 involved total protein and the examined variables and Model 2 included the interaction between total protein concentration and examined variable. Moreover, determination coefficients (R^2^) and their changes (ΔR^2^), when the interaction term was incorporated, are given.

**Figure 2:**
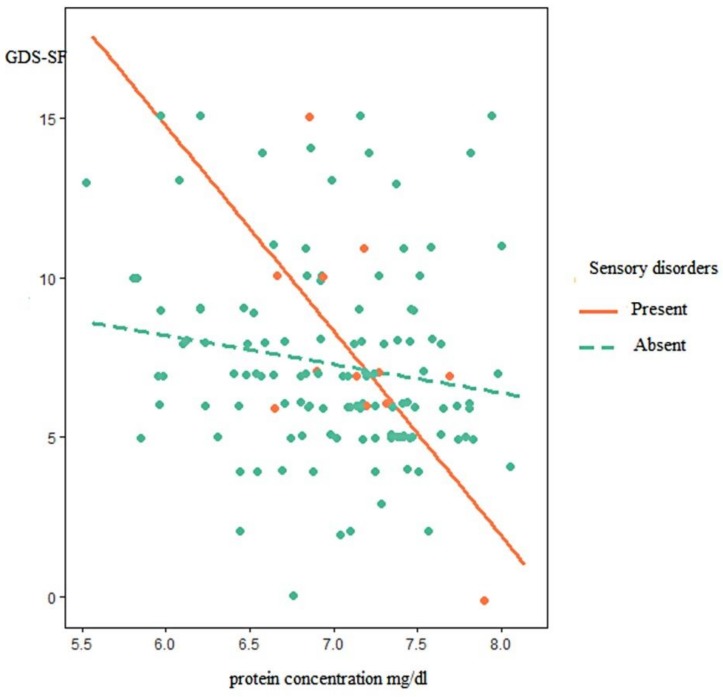
The relationship between GDS-SF and total protein concentration among subjects with and without diseases of sensory organs.

Among the comorbidities, sensory disorders significantly affected the relationship between the total protein concentration and the risk of depression occurrence, estimated using the GDS-SF scale; increasing a part of the explained variability to 8% (ΔR^2^ = 0.035) (Figure 2). Although protein was significantly related to depression (disorders (b = −6.42, 95% CI −11.27 to −1.58)), the degree of sensory disorders was not related to depression (b = −11.95, 95% CI −21.67 to −2.21). More importantly, sensory disorders moderated the relationship between protein and depression, such that the relationship was stronger among participants with sensory disorders (b = −39.7, 95% CI 23.75 to −162.29) than among participants without sensory disorders. In the case of the other analyzed diseases, no significant moderation effect was observed.

### 3.4. Mediation Model

Table 4 shows the result of testing the mediation effect using models of linear regression. Multiple regression was used to assess whether laboratory parameters, anthropometric parameters, and the results of the Barthel index were mediators of the relationship between total protein concentration and the assessment of the risk of depression occurrence (GDS-SF). Among the examined patients, the indirect effect of 25(OH)D vitamin concentration (b = −0.162, 95 % CI −0.452 to −0.015) was significant, as it was for the Barthel index (b = −0.22, 95% CI −0.54 to −0.05). In both cases, the direct effect was statistically insignificant (because there was no statistically significant relationship between total protein concentration and GDS-SF, after controlling for 25(OH)D vitamin (coefficient for total protein concentration: b = −0.88, 95% CI −1.87 to 0.11), as well as after controlling for values of the Barthel index (coefficient for total protein concentration: b = −0.79, 95% CI −1.78 to 0.21) (direct effect). Therefore, we can speak about full mediation of the relationship between GDS-SF and total protein concentration through 25(OH)D vitamin and the Barthel index. This means that the relationship between total protein concentration and the GDS-SF is transmitted through assessment of the Barthel index and 25(OH)D vitamin (mediator). Therefore, 25(OH)D vitamin and the Barthel index mediated the relationship between depression and total protein concentration.

## 4. Discussion

In our study, we showed that the significant predictors of depression included: total serum protein concentration, Barthel index, HDL and MUAC. The main result of our study was the conclusion that, in the elderly receiving long-term care, the presence or absence of sensory organ disorders significantly changed the strength of the relationship between total serum protein concentration and depression (a much stronger relationship occurred among people with these diseases). Moreover, we showed that the following variables were the mediators of the tested relationship: Barthel index and concentration of 25(OH)D vitamin. Our study results concern a certain group of patients from an older age group with physical impairment on the Barthel index (0–85 points) with comorbidities, requiring the assistance of carers (both professional and informal) concerning ADL at home, with a medium time span of 3.91 (SD = 2.61).

Evidence suggests that people of geriatric age need more protein intake in their diet than younger people. In the elderly, three groups of factors affect the level of protein in the body: insufficient protein intake (e.g., loss of appetite and gastrointestinal disorders), reduction in the body’s ability to use protein consumed with food (e.g., insulin resistance, protein anabolic resistance, and immobility) and greater demand for protein (e.g., inflammatory diseases). In this age group, the protein content in the body is of key importance in recovering from illness and maintaining functionality. Experts recommend an average daily intake of protein in the range of 1.0–1.2 g of protein per kilogram of body weight, and for physically active people (performing endurance and resistance exercises) recommendations include an intake ≥ 1.2 g/kg body weight of protein throughout the day. The protein content of 1.2–1.5 g/kg body weight per day is recommended for older people with acute or chronic diseases. However, restrictions on protein intake should apply to older people with severe renal impairment (i.e., estimated GFR < 30 mL/min/1.73 m^2^) not undergoing dialysis [36]. Physical function impairment and related hypokinesis is reflected in reaction disorders in physiological and biochemical reactions of the human organism. Ferrando et al. [14] showed that the loss of protein in the organism, caused by a lack of physical activity connected with patients who are long-term bed-ridden, is mainly the result of a decrease in muscle protein synthesis, and that this decrease is reflected both in the systemic parameters and in skeletal muscles. The decrease in protein synthesis is connected with a corresponding decrease in the sum of intracellular amino-acid composition connected with protein decomposition and internal transport. The literature shows that studies evaluating this group of patients are scarce; however, results concerning relationships between specific proteins and the occurrence of depression have been documented [37,38,39]. However, research is expensive and cannot always be performed in long-term care settings with patients with physical dysfunction. However, the assessment of functional efficiency on the Barthel index and vitamin 25(OH)D concentration in long-term care patients is possible and easy to perform in long-term care home care conditions. Our findings demonstrate that these variables are associated with depression (GDS-SF), which has been predicted by the World Health Organization to be the second-largest cause of disability worldwide by 2020, after ischemic heart disease. It is also expected to be the main cause of disability worldwide by 2030 [40]. Furthermore, in our study, the mediation and moderation effect of relationships between total protein concentration and GDS-SF were tested using PROCESS Macros for SPSS, which allows for a more advanced procedure and test to be performed, when compared with traditional methods. The significance of the moderation effect was assessed by the R square increase due to interaction, and gives the effect of moderation beyond the main effects. Similarly, to test the significance of the mediation effect, the bootstrapping approach was used.

The demonstration that the assessment of symptoms of depression is related to the concentration of total protein in the blood (Table 4) confirms, among others, study observations of protein in plasma and their observed changes in peripheral levels. This may partly reflect changes in the brain, leading to speculation on the physiological role of identified markers in the central nervous system [41]. Nevertheless, confirmation of these factors in cohort studies is required.

The theory termed by these authors *Social Signal Transduction Theory of Depression* connects psychological factors (psycho-social), immune system response, and depression. Depression is connected with the emergence of a chronic state of immune response of moderate strength, and compensative activation of the system countering the inflammation (CIRS, *anti-inflammatory reflex system*) [42]. This is accompanied by increased oxidative stress, and creation of antibodies aimed at proteins, which are modified as a result of this action [43,44,45].

In the clinical practice of psychiatric care and in home family care, knowledge of the particular types of cells and proteins engaged in immune response is not a matter of daily practice, and examination focusing on the levels of particular pro-inflammatory cytokines is unlikely to become a standard for the early diagnosis of depression. Our study showed that a simple test of total protein of blood serum in patients with physical function impairment can significantly affect the diagnosis of early depression symptoms, and the moderating factor of this correlation might also be the level of 25(OH)D vitamin.

A frequent reason for vitamin D deficiency is a decrease in the synthesis of active forms of vitamin D in kidneys. This is a physiological phenomenon concerning older adults, but also people with mobility disorders and frequently suffering from kidney disorders. Additionally, these people avoid exposition to sunlight [46]. The receptor for vitamin D is located in the brain; its deficiency not only impairs mental functions, but also the development of selected psychiatric disorders, such as schizophrenia and depression [47,48]. Zanetidou et al. [49] believed that vitamin D supplementation in older patients with coexisting dementia can result in a decrease in depression symptoms.

In the study of the possible mediating effect of depression symptoms (GDS-SF M = 3.5; SD = 3.2), correlations between ADL results (M = 96.2, SD = 8.1) and quality of life (QOL) in ambulatory community of older adults over the age of 65 (*n* = 490), it has been discovered that depression is the mediator of the relationship between the modified Barthel index, and the physical, psychological and environmental quality of life (WHOQOL-BREF) [50]. As our study concerned patients with impaired physical function (the Barthel index M = 43.20; SD = 27.06), and a higher intensity of depressive disorders (M = 7.34; SD = 3.1), it showed a slightly different model of mediation effect, with the involvement of, at least, two similar variables (depression and function impairment).

The initial conclusion is that the mediation effect can be true and deserves further confirmation. This discovery may suggest that in older adults with physical function impairment in long-term domiciliary care, the risk of depression development might be controlled, if the simple biochemical blood parameters, such as total protein and 25(OH)D vitamin, could be appropriately managed in the clinical practice of long-term care.

### 4.1. Implications of the Present Findings

The study also has implications for policy concerning people with physical function impairment under care in home conditions. Our research results indicate that people suffering from diseases of sensory organs should pay special attention to total protein concentration, which is strongly associated with the risk of depression in this group. Long-term domiciliary care patients should be systematically monitored for 25(OH)D and assessed using the Barthel index. As we have demonstrated, both variables transmitted a relationship between total protein concentration and depression. Depression symptoms impact significantly upon the quality of life of both the patient and the informal carer, including the environment of care provision and social relations. This discovery means that there remains a need for public effort in every possible regard to effectively manage the risk of the occurrence of depression in this group of patients.

### 4.2. Limitations

The current study was limited in a number of ways. The first and most significant limitation of our research is that we did not evaluate the content of protein and other ingredients in the diet. According to scientific evidence [36], adequate intake of proteins in the group of geriatric patients is very significant for maintaining efficient functionality. Second, the participants were intentionally recruited from communities with long-term care, rather than being selected randomly. This cross-sectional study was limited in its ability to make causal inferences. Thirdly, the results of the studies are based on a relatively small sample group, which might also affect the generalization of the obtained study results. Fourth, in our sample, the subjects were characterized by low and average values on the Barthel index. It is recommended to conduct a large population study including patients with varying degrees of functional capacity dysfunction (determined by the Barthel index). Such a study should also be multi-stage and longitudinal, to enable the testing of our results depending on the severity of depression symptoms. In addition, future research should include careful observation regarding protein intake in the diet.

## 5. Conclusions

Older adults with chronic diseases and physical function impairment under long-term domiciliary care experience depression. Several variables in the current study were negatively related to depression, including the levels of total protein in blood serum, and the mediators are probably low physical function (assessed by the Barthel index), and the level of 25(OH)D vitamin. Interestingly, sensory disorders moderated the relationship between total protein concentration and depression. Moreover, low physical functionality and low level of 25(OH)D vitamin reduced the relationship with total protein concentration. A cohort study is suggested to confirm the results in the current study. In addition, future research should include careful observation regarding protein intake in the diet. In conclusion, screening of simple laboratory blood indicators and their control may reduce the risk of depression in people with functional deficits.

## Figures and Tables

**Table 1:** Characteristics of the examined group.

Variable	*n* = 132
Medical interview	
Under long-term care (years)	3.91 ± 2.61 ^a^
Result on the Barthel index	43.20 ± 27.06 ^a^
Result on GDS-SF scale	7.34 ± 3.10 ^a^
Amount of medicinal substances taken	7.9 ± 2.8 ^a^
Comorbidities	
Respiratory system	19 (14.4) ^b^
Endocrine system	54 (40.9) ^b^
Nervous system	35 (26.5) ^b^
Sensory	12 (9.1) ^b^
Psychiatric	55 (41.7) ^b^
Rheumatic	77 (58.3) ^b^
Laboratory test results	
Total protein (g/dL)	7.09(6.61–7.37) ^c^
25(OH)D vitamin(ng/mL)	14.41 (8.64–26.63) ^c^
B12 vitamin (pg/mL)	390.76 ± 143.35 ^a^
Folic acid (ng/mL)	5.4 (3.74–9.92) ^c^
Total cholesterol (mg/dL)	183 (158.0–218.0) ^c^
HDL Cholesterol (mg/dL)	53.55 (42.62–64.22) ^c^
LDL Cholesterol(mg/dL)	106.5 (86.0–130.75) ^c^
Triglycerides (mg/dL)	115 (88.25–158.75) ^c^
Anthropometric variables	
Calf circumference (cm)	36.05 ± 5.42 ^a^
Mid-upper arm circumference(cm)	30.79 ± 7.90 ^a^
Tibial bone length (cm)	48.02 ± 5.44 ^a^
Thickness of the skinfold under the shoulder (cm)	2.6 (2.1–4.0) ^c^
Skinfold of arm muscle triceps (cm)	2.3 (2.0–3.8) ^c^

Data are presented as: ^a^, mean ± SD; ^b^, *n* (%); ^c^, median (Q1–Q3).

**Table 2:** Analysis of the linear regression of the depression risk (GDS-SF) determinant in the examined group.

Variable	b	95% CI
Gender		
Male	0.40	(−0.89; 1.70)
Age	0.02	(−0.04; 0.09)
Marital status		
In a relationship	−0.86	(−1.97; 0.24)
Cohabitant		
None	0.75	(−0.54; 2.03)
Medical interview		
Under long-term healthcare (years)	−0.04	(−0.26; 0.17)
Results in the Barthel index	−0.02	(−0.04; −0.004) *
Amount of medicinal substances taken	0.004	(−0.36; 0.36)
Comorbidities:		
Respiratory system	−1.45	(−2.95; 0.06)
Endocrine system	−0.33	(−1.42; 0.76)
Nervous system	0.23	(−0.98; 1.44)
Sensory	−0.36	(−2.22; 1.50)
Psychiatric	−0.35	(−1.42; 0.72)
Rheumatic	0.51	(−0.58; 1.60)
Laboratory tests results		
Total protein	−1.10	(−2.08; −0.13) *
25(OH)D Vitamin	−0.008	(−0.02; 0.007)
B12 Vitamin	−0.001	(−0.004; 0.003)
Folic acid	0.001	(−0.05; 0.06)
Total cholesterol	−0.004	(−0.01; 0.007)
HDL Cholesterol	−0.03	(−0.07; −0.002) *
LDL Cholesterol	0.001	(−0.01; 0.01)
Triglycerides	−0.001	(−0.01; 0.009)
Anthropometric variables		
Calf circumference	−0.08	(−0.18; 0.02)
Mid-upper arm circumference	−0.079	(−0.15; −0.01) *
Tibia bone length from foot base to the knee	−0.003	(−0.10; 0.10)
Thickness of under the shoulder skinfold	0.05	(−0.007; 0.12)
Skinfold of arm triceps muscle	0.06	(−0.008; 0.12)

b, coefficient from linear regression. Statistical significance is indicated by * *p* < 0.05.

**Table 3:** Moderation effect tested for the correlation between depression and protein concentration.

Model	Moderator Coefficient (95% CI)	Protein Coefficient (95% CI)	Interaction Coefficient	F
Gender				
R^2^ = 0.04	2.28 (−13.40; 17.96)	−0.77 (−3.75; 2.21)	0.27 (−2.50; 1.97)	1.81
ΔR^2^ = 0.0004	0.42 (−0.86; 1.69)	−1.11 (−2.09; −0.13)		2.71
Age				
R^2^ = 0.037	0.06 (−56.86; 77.60)	−0.44 (-9.94; 9.06)	−0.008 (−0.12; 0.11)	1.66
ΔR^2^ = 0.0001	0.0002 (−0.065; 0.065)	−1.1 (−2.15; −0.056)		2.49
Comorbidities				
Respiratory system				
R^2^ = 0.06	−0.67 (−18.78; 17.43)	−0.89 (−5.73; 3.95)	−0.10 (−2.69; 2.48)	2.86
ΔR^2^ = 0.000	−1.41 (−2.89; 0.08)	−1.09 (−2.005; 0.12) *		4.33
Endocrine system				
R^2^ = 0.05	−8.34 (−22.14; 5.46)	−2.84 (−5.97; 0.28)	1.17 (−0.80; 3.14)	2.16
ΔR^2^ = 0.01	−0.18 (-1.26; 0.90)	−1.08 (−2.07; −0.10)		2.55
Nervous system				
R^2^ = 0.04	6.86 (−9.29; 23.01)	0.51 (−3.72; 4.73)	−0.93 (−3.26; 1.39)	2.01
ΔR^2^ = 0.005	0.39 (−0.81; 1.59)	−1.14 (−2.13; −0.16) *		2.71
Sensory				
R^2^ = 0.075	−39.7 (−74.85; −4.55)	−11.95 (−21.69; −2.21) *	5.52 (0.58; 10.47) *	3.44
ΔR^2^ = 0.035 *	−0.49 (−2.33; 1.35)	−1.12 (−2.10; −0.14) *		2.64
Psychiatric				
R^2^ = 0.043	0.09 (−13.69; 13.87)	−1.04 (−4.33; 2.24)	−0.05(−2.01; 1.92)	1.89
ΔR^2^ = 0.000	−0.24 (−1.29; 0.82)	−1.12 (−2.08; -0.16) *		2.86
Rheumatic				
R^2^ = 0.046	1.59 (−12.31; 15.49)	−0.93 (−3.92; 2.07)	−0.14 (−2.12; 1.83)	2.05
ΔR^2^ = 0.0002	0.58 (−0.49; 1.64)	−1.13 (−2.11; −0.16) *		3.08

R^2^, coefficient of determination; ΔR^2^, increase R^2^ due to interaction; F, test static from model. Statistical significance is indicated by * *p* < 0.05.

**Table 4:** Mediation effect tested for correlation between depression and protein concentration.

	Mediator (M) ^A^ (Path b)		Direct Effect (Path c’) ^B^ of Protein→ GDS-SF		Indirect Effect (a*b) of Protein→M→GDS-SF	
	b	95% CI	b	95% CI	b	95% CI
Laboratory parameters:						
25 (OH)D vitamin	−0.05	(−0.10; −0.007) *	−0.786	(−1.78; 0.21)	−0.162	(−0.45; −0.01) *
B12 vitamin	−0.0003	(−0.004; 0.003)	−1.09	(−2.12; −0.06) *	−0.012	(−0.31; 0.13)
Folic acid	0.045	(−0.05; 0.14)	−1.135	(−2.12; −0.15)	0.032	(−0.04; 0.23)
Total Cholesterol	−0.003	(−0.01; 0.007)	−1.078	(−2.06; −0.09) *	−0.027	(−0.25; 0.04)
HDL Cholesterol	−0.027	(−0.06; 0.006)	−0.908	(−1.91; −0.09) *	−0.197	(−0.63; 0.01)
LDL Cholesterol	0.0004	(−0.13; 0.014)	−1.10	(−2.08; −0.12) *	−0.0013	(−0.13; 0.09)
Triglycerides	0.001	(−0.008; 0.01)	−1.132	(−2.13; −0.13) *	0.027	(−0.12; 0.30)
Result on the Barthel index	−0.02	(−0.04; −0.0002) *	−0.882	(−1.87; 0.11)	−0.223	(−0.54; −0.05) *
Anthropometric variables:						
Calf circumference	−0.073	(−0.17; 0.02)	−1.039	(−2.01; −0.07) *	−0.035	(−0.35; 0.07)
Mid-upper arm circumference	−0.141	(−0.25; −0.035) *	−0.900	(−1.86; 0.06)	−0.174	(−0.50; 0.02)
Tibia bone length from foot base to the knee	−0.011	(−0.11; 0.09)	−1.082	(−2.06; −0.10) *	0.008	(−0.12; 0.24)
Thickness of under the shoulder skinfold	0.05	(−0.01; 0.11)	−1.014	(−1.99; −0.04) *	−0.059	(−0.50; 0.06)
Arm triceps muscle skinfold	0.05	(−0.01; 0.12)	−1.014	(−1.99; −0.04) *	−0.600	(−0.47; 0.07)

b, coefficient from linear regression; ^A^, association between mediator and GDS-SF after controlling for total protein concentration; ^B^, association between total protein concentration and GDS-SF after controlling for moderator; statistical significance is indicated by * *p* < 0.05.
